# The association of perceptions of harmfulness and addictiveness on the age of initiation of cigar product use among youth: Findings from the Population Assessment of Tobacco and Health (PATH) study, 2013–2017

**DOI:** 10.3389/fpubh.2022.882434

**Published:** 2022-10-05

**Authors:** Baojiang Chen, Charles E. Spells, Meagan A. Bluestein, Arnold E. Kuk, Melissa B. Harrell, Adriana Pérez

**Affiliations:** ^1^Department of Biostatistics and Data Science, School of Public Health, the University of Texas Health Science Center at Houston (UTHealth Houston), Austin, TX, United States; ^2^Michael & Susan Dell Center for Healthy Living, School of Public Health, The University of Texas Health Science Center at Houston (UTHealth), Austin, TX, United States; ^3^Department of Epidemiology, School of Public Health, Human Genetics and Environmental Sciences, The University of Texas Health Science Center at Houston (UTHealth Houston), Austin, TX, United States

**Keywords:** cigarillos, filtered cigars, traditional cigars, time to event, interval censoring, age of onset, large cigars, little cigars

## Abstract

**Background:**

Perceptions of cigar products' harmfulness and addictiveness in youth are associated with subsequent cigar product initiation, but their association on the age of initiation of cigar product use is unknown.

**Methods:**

The association of perceptions of harmfulness and addictiveness at youth's first wave of PATH participation (waves 1 or 2 in years 2013–2015) on the age of initiation of (i) ever. (ii) past 30-day, and (iii) fairly regular use of any cigar products (cigarillos, filtered cigars, or traditional cigars) during the followed-up in PATH waves 2–4 (2014–2017) was estimated using weighted interval-censored Cox proportional hazards models. Also, the association of the interaction between perceptions of harmfulness and addictiveness and the age of initiation of any cigar use are reported. Hazard ratios (HR) and 95% confidence intervals (CI) are reported.

**Results:**

Among youth who had ever heard of cigar products, youth who perceived cigars to be “low-medium harmfulness and low-medium addictiveness” had 60% (HR: 1.60, 95%CI: 1.36–1.89) higher hazard risk to initiate ever cigar product use at an earlier age, and had 46% (HR:1.46, 95%CI: 1.14–1.86) higher hazard risk to initiate past 30-day cigar product use at younger ages than those who perceived cigars to be “high harmfulness and high addictiveness.” Moreover, youth who perceived cigars to be “low-medium harmfulness and high addictiveness” had 33% (HR: 1.33, 95%CI: 1.15–1.53) higher hazard risk to initiate ever cigar product use at younger ages than those who perceived cigars to be “high harmfulness and high addictiveness.” Youth who reported “high harmfulness and low-medium addictiveness” (HR: 0.24, 95% CI: 0.07–0.83) had 76% lower hazard risk to initiate fairly regular use of cigar products at younger ages compared to youth who reported “high harmfulness and high addictiveness.”

**Conclusions:**

Prevention and awareness campaigns should reinforce the unique potential for harm and addiction of cigar products to curb cigar product initiation among US youth.

## Introduction

Lower perceptions of harmfulness and addictiveness of tobacco products in youth is a known predictor of subsequent tobacco use behaviors (1). Previous research shows that youth who perceived cigarettes as having “low” harm were more than 2 times more likely to initiate cigarette use than those who held the highest harm perceptions (2). However, information on the association of perceptions of harmfulness and addictiveness of cigar products on cigar product initiation is limited. The 2019 National Youth Tobacco Survey (NYTS) found cigar products (traditional cigars, cigarillos, and little cigars) to be the most commonly used combustible tobacco product among middle and high school students (7.6% of high schoolers and 2.3% of middle schoolers), surpassing cigarettes (5.8% of high schoolers and 2.3% of middle schoolers) (3).

One study using the 2012–2014 NYTS showed that roughly one-third of youth (grades 6–12) believed that cigars products (cigars, cigarillos, or little cigars) were less addictive than cigarettes (1), and 25.8% of youth viewed cigars as less harmful than cigarettes (1). Data from 2013-2015 of the Population Assessment of Tobacco and Health (PATH) study found that youth who perceived cigar products as having “low” harmfulness were more likely to try traditional cigars, cigarillos, and filtered cigars (*p* = 0.001–0.014) 1 year later when compared to youth who perceived each cigar product as having “high” harmfulness (4). This same study found that youth who perceived cigar products as having “low” addictiveness were more likely to try traditional cigars, cigarillos, and filtered cigars (*p* = 0.001) 1 year later when compared to youth who perceived each cigar product as having “high” addictiveness (4).

A lack of understanding of the possible health harms and addiction potential associated with cigar product use is problematic because cigar products contain nicotine and other harmful toxicants and can be detrimental to health in quantities that actually surpass cigarettes (5–7). For example, traditional cigars can deliver ten times the nicotine and twice the amount of tar compared to cigarettes (7); cigarillos contain 5–12 times as much nicotine as cigarettes (5); and filtered cigars contain higher carbonyl levels compared to cigarettes (6). Additionally, the use of cigar products has been linked to COPD, asthma, and high blood pressure (8). Early onset of cigar product use at younger ages also increases the risk for addiction to nicotine (7, 9). Nicotine content of cigars ranges from 10 to 444 mg depending on the weight of the cigar (10). By comparison, the nicotine content of cigarettes ranges from 6–29 mg (11). These negative health effects are particularly troubling, given that cigar products have become more popular than cigarettes among youth.

Earlier age of initiation of cigar product use at younger ages may exacerbate substantial harm to health (7, 9, 12). The age of initiation of cigar use behaviors is important to understand as we begin to identify when youth groups are most vulnerable to cigar initiation and which factors are associated with the age of initiation of cigar product use. Little research has been conducted to identify modifiable risk factors that decrease the age of initiation of cigar product use, including the perceptions of harmfulness and addictiveness. The relationship between the perceptions of harmfulness and addictiveness on the initiation of cigarette use has been studied in youth (1, 2). However, the association of perceptions of harmfulness and addictiveness on the age of initiation of cigar use has not been explored, which is important for prevention efforts. This study examines youth (12–17) never users of cigar products using waves 1–4 of the PATH study to determine the association of perceptions of harmfulness and addictiveness on the age of initiation of three cigar product use outcomes: ever, past 30-day and fairly regular use. The use of cigarillos, filtered cigars, and traditional cigars were combined to represent any cigar product use. We hypothesized that youth with low or medium perceptions of harmfulness and addictiveness of cigar products had higher hazard of initiating of ever, past 30-day or fairly regular use of any cigar product at an earlier age compared to youth with high perceptions of harmfulness and addictiveness of cigar products.

## Materials and methods

### Study design and participants

PATH is a U.S. nationally representative study of youth (12–17 years old) and adults (18+ years old) that measures the use of tobacco products since 2013 and the design of the study is reported elsewhere (13). Four waves of PATH restricted data were available at the time of analysis: wave 1: 2013–2014, wave 2: 2014–2015, wave 3: 2015–2016, wave 4: 2016–2017. Parents provided informed consent and youth provided verbal assent. IRB approval for this study was obtained from the Committee for the Protection of Human Subjects at the University of Texas Health Science Center at Houston with number HSC-SPH-17–0368.

In this study, a cohort of participants who were 12–17 years old and had never used any cigar product at their first wave of PATH participation in waves 1 or2 (2013–2015) were included in the analysis, as the harmfulness and addictiveness questions were only asked at PATH waves 1 and 2 for cigar products. These participants were followed in PATH waves 2–4 (2014–2017) to measure their age of initiation of each cigar outcome. There were 12,261 youth who had never used any cigar product and entered the PATH study at wave 1, and there were 2,070 additional youth who had never used any cigar product entered the PATH study at wave 2. The sample for this study includes participants who had heard or had not heard of or answered “don't know” for traditional cigars, cigarillos or filtered cigars at the first wave of PATH participation. We used this sample to study the main effect of the perceptions of harmfulness and addictiveness on the age of initiation of each cigar use outcome, respectively.

We further used a subsample (*n* = 8,561) of youth who had heard of these cigar products to study the interaction effect between the perceptions of cigar product harmfulness and addictiveness on the age of initiation of each cigar use outcome. In this subsample, 7,235 youth who had never used any cigar product entered the study at wave 1, and 1,326 shadow youth never cigar product users entered the study at wave 2. Shadow youth were family members of PATH participants who were 9–11 years old at wave 1, and once they reached 12 years old they were invited to participate in the PATH study.

### Measures

#### Outcomes: Ever, past 30-day and fairly regular use of any cigar product

In waves 1–4, PATH asked participants “Have you ever smoked a [TP], even one or two puffs?” (14), where [TP] includes traditional cigars, cigarillos, or filtered cigars. There was a total of 14,331 never cigar youth users at the first wave of PATH participation who had their outcomes followed–up in waves 2–4 (2014–2017). Participants who reported “yes” to any of three products were categorized as ever users of any cigar in waves 2–4. Similarly, PATH asked participants “In the past 30 days, on how many days did you smoke a [TP]?”. Participants who reported using any of the three cigar products in the past 30 days for the first time in waves 2–4 were categorized as past 30–day users of any cigar product. PATH asked participants “Have you ever smoked [TP] fairly regularly?”. Participants who reported “yes” to any of three cigars products for the first time in waves 2–4 were categorized as fairly regular users of any cigar product. Youth who answered “don't know” or “refused” to any of the questions above were categorized as missing in waves 2–4 and were also included in the analysis.

#### Exposures: Perceptions of harmfulness and addictiveness

We examined perceptions of harmfulness and addictiveness at youths' first wave of PATH participation on the age of initiation of each cigar product, as these perceptions of harmfulness and addictiveness were only available at waves 1 and 2. The following question was used to measure perceptions of harmfulness of any cigar product among those who had heard of traditional cigars, cigarillos or filtered cigars: “How much do you think people harm themselves when they smoke traditional cigars, cigarillos or filtered cigars?” (14). Response options included (i) “No harm”; (ii) “Little harm”; (iii) “Some harm”; (iv) “A lot of harm”; (v) “Don't know”; and (vi) “Refused”. The answers “No harm” or “Little harm” were recoded as low perception of harmfulness, “Some harm” was recoded as medium perception of harmfulness, and “A lot of harm” was recoded as high perception of harmfulness.

The following question was used to measure perceptions of addictiveness of any cigar product among those who had heard of traditional cigars, cigarillos or filtered cigars: “How likely is someone to become addicted to traditional cigars, cigarillos or filtered cigars?” (14). Response options included (i) “Very unlikely”; (ii) “Somewhat unlikely”; (iii) “Neither likely nor unlikely”; (iv) “Somewhat likely”; (v) “Very likely”; (vi) “Don't know”; and (vii) “Refused.” The answers “Very unlikely” or “Somewhat unlikely” were recoded as low perception of addictiveness, “Neither likely nor unlikely” was recoded as medium perception of addictiveness, and “Somewhat likely” or “Very likely” was recoded as high perception of addictiveness.

For both questions, those who answered “don't know” were kept as a category in our analysis, and we excluded youth who “refused” to answer these questions. Both the perceptions of harmfulness and addictiveness questions were not asked among those who had not heard of traditional cigars, cigarillos or filtered cigars, thus we created a new category “Never heard of cigars” in our analysis to maximally use available data.

Further, we used the subsample of those participants who had heard of traditional cigars, cigarillos or filtered cigars to study the interaction effect between perceptions of harmfulness and addictiveness. We further created a variable to represent the interaction of harmfulness and addictiveness at participants' first wave of PATH participation, which was based on a dichotomized combination of the perceptions of harmfulness and addictiveness. The answers “Low” and “Medium” harmfulness was combined and recoded as “Low–Medium,” and the “High” harmfulness category as reference category. This same combination was made for addictiveness. We collapsed these variables based on the distributions of harmfulness and addictiveness, because the hazard ratios of cigar use for the categories of “Low” and “Medium” levels were similar but different from the “High” category. In addition, an interaction term of 2x2 is easier to interpret than a 3x3. The interaction of harmfulness and addictiveness resulted in four categories “high harm and high addictiveness,” “high harm and low–medium addictiveness,” “low–medium harm and high addictiveness,” and “low–medium harm and low–medium addictiveness.”

### Other tobacco product use

We controlled for ever use of other tobacco products at youths' first wave of PATH participation to ensure that other tobacco product use preceded any cigar product initiation. In addition to cigar product use, PATH also measured ever use of other tobacco products using the question “Have you ever smoked a [TP], even one or two puffs?”. These products included cigarettes, e–cigarettes, hookah, and smokeless tobacco. A new variable for the number of other tobacco products used was created with values 0 (never used other tobacco products), 1 (used one other tobacco product), and 2+ (used 2 or more other tobacco products).

### Sex and race/ethnicity

Sex classified youth as males or females and PATH in 2013–2014 imputed this variable using the household information but not at subsequent waves. In PATH, race was assessed as White race alone, Black race alone, Asian race alone, and other race (including multi–racial), and ethnicity was categorized as either Hispanic or Non–Hispanic. To be comparable to the Surgeon General's reports (15, 16), we classified race/ethnicity into four categories: Non–Hispanic White, Hispanic, Non–Hispanic Black, Non–Hispanic Other (Asian, multi–race, and other races).

### Interval–censored age of initiation of any cigar product use

The age of initiation of ever, past 30–day, or fairly regular use of any cigar product is defined as the age of initiation of ever use of any cigar product, and the age of first report of past 30–day or fairly regular use of any cigar product, respectively. The exact date of initiation of each outcome (ever, past 30–day, and fairly regular use) was not asked in PATH. Participants' birthdays are also not included in the restricted–use dataset. To estimate a lower and upper age bound for each cigar use outcome, we used 2 variables: (i) the participant's age (in years) at the first wave of PATH youth participation and (ii) the number of weeks between survey waves. For all participants, the lower bound was the age at the last wave where the participant reported non–use of each cigar outcome. For those who became users, the upper bound was the lower bound age (i.e., the age where they are non–users) plus the number of weeks between survey waves that the participant first reported initiation of each cigar outcome. For those who remained non–users, the upper age bound was censored.

### Statistical analysis

All analyses conducted incorporate sampling weights and the balance repeated replicate weights with Fay's factor of 0.3 to account for the complex survey design in PATH (17). Weighted summary statistics, including means and standard errors for continuous variables, and frequencies and percentages for categorical variables, are reported. The interval–censored Cox proportional hazards models with the piecewise constant baseline hazard function (18) were used to explore the association of perceptions of harmfulness and addictiveness, separately, on the age of initiation of each outcome while adjusting for sex, race/ethnicity and the number of other tobacco products ever used. The “high perception of harmfulness” and “high perception of addictiveness” were used as reference categories, respectively. These analyses were conducted on a sample of youth never cigar product users that included youth who had never heard of cigar products. We also conducted an analysis only on the subsample of youth who had heard of cigar products to examine the association of the interaction of perceptions of harmfulness and addictiveness on the age of initiation of each outcome while adjusting for sex, race/ethnicity and the number of other tobacco products ever used. The “high perception of harmfulness and high perception of addictiveness” was used as a reference category. The crude and adjusted hazard ratios (AHRs) and their 95% confidence intervals (CIs) are reported. Missing data are reported for covariates. All statistical analyses were completed in SAS version 9.4 using the Inter–university Consortium for Political and Social Research server hosted by the University of Michigan.

## Results

### Main effects of perceptions of harmfulness and addictiveness on the age of initiation of cigar product use

Table 1 describes *n* = 14,331 youths, representing *N* = 26,390,886 U.S. youth who had never used any cigar product at their first wave of PATH participation (2013–2015). Among these participants, about 85% entered the PATH study at wave 1 (2013–2014), their mean age was 14.0 (SE = 0.01), 50% of participants were male and 87% of our sample had never used any other tobacco products. In terms of race/ethnicity, 23% were Hispanic, 54% were Non–Hispanic White, 14% were Non–Hispanic Black, and 10% were Non–Hispanic other race/ethnicity. Among all participants, 4% reported perceptions of harmfulness as low harm, 19% reported perceptions of harmfulness as medium harm, and 39% reported perceptions of harmfulness of cigar products as high harm. Among all participants, 5% reported perceptions of addictiveness as low addiction, 6% reported perceptions of addictiveness as medium addiction, and 50% reported perceptions of addictiveness high addiction of cigar products. About 37% youth had not heard of any cigar product.

**Table 1:** Sociodemographic characteristics of PATH US youth (aged 12–17) who were never cigar users at their first wave of participation 2013–2015 (*n* = 14,331, *N* = 26,390,886)^*^.

**Variables**	**Unweighted frequency**	**Weighted frequency**	**Weighted percent (SE)**
**Wave of entry into PATH**			
Wave 1	12,261	22,319,081	84.6 (0.10)
Wave 2	2,070	4,071,805	15.4 (0.10)
**Sex**			
Male	7,186	13,221,810	50.1 (0.14)
Female	7,141	13,161,160	49.9 (0.14)
Missing	4	7,916	
**Age at entry into study** **(mean, SE)**	14.0 (0.01)		
**Race/ethnicity**			
Hispanic	4,159	6,021,896	22.8 (0.13)
Non–Hispanic White	6,902	14,183,640	53.8 (0.15)
Non–Hispanic Black	1,941	3,637,424	13.8 (0.12)
Non–Hispanic Other^†^	1,315	2,523,869	9.6 (0.12)
Missing	14	24,057	
**Perception of harmfulness**			
Low	640	1,158,834	4.4 (0.18)
Medium	2,713	4,986,567	18.9 (0.35)
High	5,531	10,317,081	39.1 (0.49)
Don't know	126	240,636	0.9 (0.10)
Never heard	5,321	9,687,768	36.7 (0.45)
**Perception of addictiveness**			
Low	691	1,249,573	4.7 (0.17)
Medium	875	1,597,199	6.1 (0.22)
High	7,057	13,122,414	49.7 (0.50)
Don't know	387	733,932	2.8 (0.14)
Never heard	5,321	9,687,768	36.7 (0.45)
**Number of other tobacco products ever used**			
0	12,439	22,954,247	87.0 (0.35)
1	1,162	2,094,388	7.9 (0.24)
2+	730	1,342,252	5.1 (0.22)

* PATH Restricted file received disclosure to publish: April 22, 2021. United States Department of Health and Human Services. National Institutes of Health. National Institute on Drug Abuse, and United States Department of Health and Human Services. Food and Drug Administration. Center for Tobacco Products. Population Assessment of Tobacco and Health (PATH) Study [United States] Restricted–Use Files. ICPSR 36231–v13.AnnArbor, MI: Inter–university Consortium for Political and Social Research [distributor], November 5, 2019. https://doi.org/10.3886/ICPSR36231.v23.

† Non–Hispanic Others include Asian, multi–race, etc.

Table 2 reports the crude and adjusted hazard ratios and 95% CIs exploring the association of perceptions of harmfulness and addictiveness, separately, on the age of initiation of each cigar use outcome while controlling for sex, race/ethnicity and the number of other tobacco products ever used (See Supplementary Table 1 for the entire models). Compared to youth who reported high perceptions of harmfulness, youth who reported low perceptions of harmfulness had 87% (HR: 1.87, 95% CI: 1.56–2.25) higher hazard ratio to initiate ever use of any cigar product at earlier ages, 83% (HR: 1.83, 95% CI: 1.46–2.29) higher hazard ratio to initiate past 30–day use of any cigar product at earlier ages, and 108% (HR: 2.08, 95% CI: 1.01–4.30) higher hazard ratio to initiate fairly regular use of any cigar product at earlier ages. Youth who reported medium perceptions of harmfulness had 26% (HR: 1.26, 95% CI: 1.11–1.43) higher hazard ratio to initiate ever use of any cigar product at earlier ages compared to those with high perceptions of harmfulness. Youth who had not heard of any cigar product had decreased risk of initiating ever use of any cigar product at earlier ages (HR: 0.71, 95% CI: 0.63–0.80) and past 30–day use of any cigar product (HR: 0.72, 95% CI: 0.61–0.85) at younger ages compared to those with high perceptions of harmfulness.

**Table 2:** Hazard ratio (and 95% confidence intervals) of perceptions of harmfulness and addictiveness for each cigar outcome^*^.

**Outcome**	**Unadjusted analysis**	Adjusted analysis^**^
**Ever use**		
**Perception of harmfulness**		
High	1.00	1.00
Low	**2.11 (1.78–2.49)**	**1.87 (1.56–2.25)**
Medium	**1.39 (1.24–1.56)**	**1.26 (1.11–1.43)**
Don't know	1.58 (0.93–2.69)	1.51 (0.92–2.47)
Never heard	**0.66 (0.59–0.75)**	**0.71 (0.63–0.80)**
**Perception of addictiveness**		
High	1.00	1.00
Low	**1.67 (1.41–1.99)**	**1.34 (1.11–1.61)**
Medium	**1.41 (1.19–1.67)**	**1.22 (1.02–1.46)**
Don't know	1.18 (0.88–1.59)	1.14 (0.85–1.54)
Never heard	**0.60 (0.54–0.68)**	**0.65 (0.57–0.73)**
**Past 30–day use**		
**Perception of harmfulness**		
High	1.00	1.00
Low	**2.10 (1.67–2.64)**	**1.83 (1.46–2.29)**
Medium	**1.21 (1.04–1.42)**	1.09 (0.92–1.29)
Don't know	1.43 (0.61–3.37)	1.37 (0.60–3.12)
Never heard	**0.67 (0.57–0.79)**	**0.72 (0.61–0.85)**
**Perception of addictiveness**		
High	1.00	1.00
Low	**1.75 (1.39–2.20)**	**1.35 (1.05–1.72)**
Medium	**1.34 (1.07–1.69)**	1.13 (0.89–1.43)
Don't know	1.28 (0.85–1.93)	1.26 (0.84–1.91)
Never heard	**0.64 (0.54–0.76)**	**0.69 (0.58–0.82)**
**Fairly regular use**		
**Perception of harmfulness**		
High	1.00	1.00
Low	**2.39 (1.16–4.94)**	**2.08 (1.01–4.30)**
Medium	1.46 (0.86–2.47)	1.35 (0.79–2.31)
Don't know	1.84 (0.28–12.26)	1.66 (0.25–11.19)
Never heard	0.53 (0.26–1.07)	0.56 (0.28–1.13)
**Perception of addictiveness**		
High	1.00	1.00
Low	1.25 (0.56–2.80)	1.00 (0.43–2.33)
Medium	0.61 (0.27–1.37)	0.53 (0.23–1.21)
Don't know	0.37 (0.03–5.10)	0.35 (0.02–4.87)
Never heard	**0.40 (0.21–0.74)**	**0.43 (0.23–0.79)**

* PATH Restricted file received disclosure to publish: April 22, 2021. United States Department of Health and Human Services. National Institutes of Health. National Institute on Drug Abuse, and United States Department of Health and Human Services. Food and Drug Administration. Center for Tobacco Products. Population Assessment of Tobacco and Health (PATH) Study [United States] Restricted–Use Files. ICPSR 36231–v13.AnnArbor, MI: Inter–university Consortium for Political and Social Research [distributor], November 5, 2019. https://doi.org/10.3886/ICPSR36231.v23.

** , adjusted for sex, race/ethnicity and number of other tobacco products used. For the perception of harmfulness, the perception of addictiveness was not included as a covariate; and for the perception of addictiveness, the perception of harmfulness was not included as a covariate. In other words, the association of these variables on the age of initiation of cigar products was considered separately. Bold values indicate statistical significant difference *p*-value < 0.05.

Compared to youth who reported high perceptions of addictiveness, youth who reported low perceptions of addictiveness had 34% (HR: 1.34, 95% CI: 1.11–1.61) higher hazard ratio to initiate ever use of any cigar product at earlier ages, and 35% (HR: 1.35, 95% CI: 1.05–1.72) higher hazard ratio to initiate past 30–day use of any cigar product at earlier ages. Youth who reported medium perceptions of addictiveness had 22% (HR: 1.22, 95% CI: 1.02–1.46) higher hazard ratio to initiate ever use of any cigar product at earlier ages compared to those with high perceptions of addictiveness. Youth who had not heard of any cigar product had lower risk to initiate ever use of any cigar product at earlier ages (HR: 0.65, 95% CI: 0.57–0.73), past 30–day use of any cigar product at earlier ages (HR: 0.69, 95% CI: 0.58–0.82) and fairly regular use of any cigar product (HR: 0.43, 95% CI: 0.23–0.79) at younger ages compared with those with high perceptions of addictiveness.

### Interaction effects of perceptions of harmfulness and addictiveness on the age of initiation of cigar product use

Table 3 reports sociodemographic characteristics of the subset of youth who had heard of any cigar product and answered the perception of harmfulness and addictiveness questions (*n* = 8,561; *N* = 15,851,230). Among these participants, almost 83% entered the PATH study at wave 1 (2013–2014), their mean age was 14.2 (SE = 0.02), 50% of participants were male and 84% of our sample had never used any other tobacco products. In terms of race/ethnicity, 20% were Hispanic, 57% were Non–Hispanic White, 15% were Non–Hispanic Black, and 8% were Non–Hispanic other race/ethnicity. Among all participants, 11% reported “low–medium harmfulness and low–medium addictiveness,” 26% reported “low–medium harmfulness and high addictiveness,” 7% reported “high harmfulness and low–medium addictiveness,” and 56% reported “high harmfulness and high addictiveness.”

**Table 3:** Sociodemographic characteristics of PATH US youth (aged 12–17) who were never cigar users at their first wave of participation 2013–2016 and who had heard of cigar products (*n* = 8,561, *N* = 15,851,230)^*^.

**Variables**	**Unweighted frequency**	**Weighted frequency**	**Weighted percent (SE)**
**Wave of entry into PATH**			
Wave 1	7,235	13,207,963	83.3 (0.29)
Wave 2	1,326	2,643,268	16.7 (0.29)
**Sex**			
Male	4,228	7,874,872	49.7 (0.40)
Female	4,333	7,976,359	50.3 (0.40)
**Age at entry into study (mean, SE)**	14.2 (0.02)		
**Race/ethnicity**			
Hispanic	2,162	3,170,724	20.0 (0.31)
Non–Hispanic White	4,338	8,943,535	56.5 (0.42)
Non–Hispanic Black	1,290	2,392,360	15.1 (0.29)
Non–Hispanic Other^†^	766	1,334,161	8.4 (0.24)
Missing	5	10,451	
**Perception of harmfulness and addictiveness**			
Low–medium harmfulness and low–medium addictiveness	969	1,774,447	11.2 (0.38)
Low–medium harmfulness and high addictiveness	2,267	4,159,615	26.2 (0.56)
High harmfulness and low–medium addictiveness	578	1,038,145	6.55 (0.29)
High harmfulness and high addictiveness	4,747	8,879,024	56.01 (0.70)
**Number of other tobacco products ever used**			
0	7,159	13,329,369	84.1 (0.46)
1	850	1,525,791	9.6 (0.32)
2+	552	996,070	6.3 (0.31)

* PATH Restricted file received disclosure to publish: April 22, 2021. United States Department of Health and Human Services. National Institutes of Health. National Institute on Drug Abuse, and United States Department of Health and Human Services. Food and Drug Administration. Center for Tobacco Products. Population Assessment of Tobacco and Health (PATH) Study [United States] Restricted–Use Files. ICPSR 36231–v13.AnnArbor, MI: Inter–university Consortium for Political and Social Research [distributor], November 5, 2019. https://doi.org/10.3886/ICPSR36231.v23.

† Non–Hispanic Others include Asian, multi–race, etc.

Table 4 reports the adjusted hazard ratios and 95% CIs exploring the interaction between perceptions of harmfulness and addictiveness on the age of initiation of each cigar outcome while controlling for sex, race/ethnicity, and the number of other tobacco products ever used among youth never cigar product users who had heard of cigar products at their first wave of PATH participation (PATH waves 1–2, 2013–2015) (See Supplementary Table 2 for the entire models). Compared to youth who reported “high harmfulness and high addictiveness” of any cigar product, youth who reported “low–medium harmfulness and high addictiveness” of any cigar product (HR: 1.33, 95% CI: 1.15–1.53), and “low–medium harmfulness and low–medium addictiveness” of any cigar product (HR: 1.60, 95% CI: 1.36–1.89) had higher hazard ratios (33% and 60%, respectively) to initiate ever use of any cigar product at younger ages. Youth who reported “low–medium harmfulness and low–medium addictiveness” of any cigar product (HR: 1.46, 95% CI: 1.14–1.86) had 46% higher hazard risk to initiate past 30–day use of any cigar product at younger ages compared to youth who reported “high harmfulness and high addictiveness.” Youth who reported “high harmfulness and low–medium addictiveness” (HR: 0.24, 95% CI: 0.07–0.83) had 76% lower hazard ratio to initiate fairly regular use of any cigar product at younger ages compared to youth who reported “high harmfulness and high addictiveness”. See Supplemental Figures 1–3 for the estimated hazard function for the age of initiation of ever, past 30–day and fairly regular cigar use stratified by perceptions of harmfulness and addictiveness.

**Table 4:** Hazard ratio (and 95% confidence intervals) of the interaction between perceptions of harmfulness and addictiveness^*^.

Perception of harmfulness and addictiveness^**^	**Ever use**	**Past 30–day use**	**Fairly regular use**
High harmfulness and high addictiveness	1.00	1.00	1.00
High harmfulness and low–medium addictiveness	1.16 (0.91–1.46)	1.07 (0.81–1.41)	**0.24 (0.07–0.83)**
Low–medium harmfulness and high addictiveness	**1.33 (1.15–1.53)**	1.15 (0.95–1.40)	1.36 (0.78–2.38)
Low–medium harmfulness and low–medium addictiveness	**1.60 (1.36–1.89)**	**1.46 (1.14–1.86)**	1.31 (0.66–2.61)

* PATH Restricted file received disclosure to publish: April 22, 2021. United States Department of Health and Human Services. National Institutes of Health. National Institute on Drug Abuse, and United States Department of Health and Human Services. Food and Drug Administration. Center for Tobacco Products. Population Assessment of Tobacco and Health (PATH) Study [United States] Restricted–Use Files. ICPSR 36231–v13.AnnArbor, MI: Inter–university Consortium for Political and Social Research [distributor], November 5, 2019. https://doi.org/10.3886/ICPSR36231.v23.

** , adjusted for sex, race/ethnicity and number of other tobacco products used. Bold values indicate statistical significant difference *p*-value < 0.05.

We further evaluated the heterogeneity of effects of perceptions of harmfulness and addictiveness on the age of initiation of each outcome among youth by comparing the sum of the independent effects of harmfulness and addictiveness (expected effect) with the observed joint effect (the interaction). Our interaction analysis found that the observed joint effect of perceptions of harmfulness and addictiveness is smaller than expected on the age of initiation of ever (expected 1.81 vs. observed joint effect 1.60) and past 30–day cigar use (expected 1.58 vs. observed joint effect 1.46), suggesting that the interaction effect is antagonistic.

## Discussion

The purpose of this study was to examine the association of youths' perceptions of harmfulness and addictiveness of cigar products on the age of initiation of cigar product use. Our findings expand on previous studies that examined how youths' perceptions are associated with cigar product initiation (4, 19, 20) by focusing on their age of initiation. Age of initiation of cigar use behaviors is important to understand as we begin to identify when youth groups are most vulnerable to cigar initiation based on how harmful or addictive they perceive cigar products to be.

This study found that youth who had never heard of cigar products had lower hazard risk to initiate cigar product use at earlier ages when compared to youth who held high perceptions of harmfulness and addictiveness of cigar products. While this phenomenon may be protective for those who had never heard of cigar products, as these products have increased in popularity among U.S. youth in recent years (21, 22), increasing young people's knowledge and awareness around the potential harmfulness and addictiveness of cigar products is necessary (19). In addition, these findings suggest that awareness of cigar products may indicate exposure to peers or family members who use cigars, cigar marketing, or other external influences that are associated with cigar initiation (4). Age of initiation is important to study because youth who initiate tobacco products earlier are more likely to continue use of tobacco products in adulthood (15). More research is needed among adult cigar product users to determine how their age of initiation is associated with their cigar product use in adulthood. As the prevalence of cigar products increases among young people, this research on the association of perceptions of cigar product harmfulness and addictiveness on cigar product use is needed to inform youth tobacco prevention efforts.

To our knowledge, this study is novel by incorporating an interaction between harmfulness and addictiveness perceptions to estimate the association of these associations on the age of initiation of cigars. Because we use a novel interaction to measure this association, we did not find similar studies to compare our results with on the association on the age of initiation; for that reason, we compare our results against the association of perceptions of harmfulness and addictiveness on initiation of cigar products separately. This study found that youth who perceive cigar products as having low–medium harmfulness and low–medium addictiveness had higher hazard to initiate ever use and past 30–day use of cigar products at earlier ages than those who perceive cigar products as having high harmfulness and addictiveness. The relationship between perceived harmfulness and addictiveness of cigar products is inversely related to the age of initiation, meaning that lower levels of perceived harmfulness and addictiveness among youth are associated with increased risk of an earlier age of initiation of cigar product use. This finding was supported in previous research, which found that perceptions of harmfulness and addictiveness in youth never users of traditional cigars, cigarillos, and filtered cigars were inversely related to trying a tobacco product for the first time 1 year later (i.e., those with lower perceptions of harmfulness and addictiveness have increased risk of initiating the cigar product) (4). The antagonistic effect of perceptions of harmfulness and addictiveness on the age of initiation of ever and past 30–day use of cigar products, while smaller than expected, suggest an overlap in the constructs of perceptions of harmfulness and addictiveness of cigar products. This implies that prevention campaigns may have equal effect or benefit more from either focusing on perceptions of harmfulness or perceptions addictiveness exclusively vs. addressing these concepts within the same campaign.

Another PATH study found that youth's low perceptions of harmfulness of cigar products increased the likelihood of having tried cigar products 1 year later (20). Although this previous study only examines the association between perceptions of harmfulness and ever use of cigar products, our findings adds to this existing literature by providing evidence on the association of lower perceptions of harmfulness and addictiveness on the age of initiation of past 30–day and fairly regular cigar product use. These findings are important as increased frequency of tobacco use increases the risk of long–term health effects and lifelong nicotine addiction (15, 23, 24).

### Strengths and limitations

In this study we use an interval–censored measurement for the age of initiation across four waves of PATH data. Given that it is unfeasible to ask youth the exact date they initiated cigar product use, this measure reduces recall bias in the age estimation by using the number of weeks between surveys to calculate a more precise measurement of age (25). This study component is not without limitation: because we prospectively examined perceptions of harmfulness and addictiveness among youth never users of cigar products, youth who initiated cigar outcomes (e.g., ever use, past 30–day use, and fairly regular use) prior to their first wave of PATH participation were excluded from this study, which could result in an underestimation of the age of initiation. Since PATH only asked youth questions about their perceptions of harmfulness and addictiveness of cigar products in waves 1 and 2, our study is limited in its ability to assess risk perceptions that may have changed over time. In this study, we only controlled for a limited set of covariates. There may be other variables that are associated with the age of initiation of cigar product use, such as socio–economic status, parents or peer use, internalization and externalization problems, exposure to tobacco advertisements, etc. To study the direct effect of the harmfulness and addictiveness and keep the model simple, we did not control for these variables. Another limitation found in our study is we did not stratify our analyses by those who use cigar products as intended from those who use cigar products as blunts [i.e., cigar products filled with marijuana (21)]. This was outside the scope of our study and should be explored in future research.

## Conclusion

This study prospectively examines the association of perceptions of harmfulness and addictiveness on the age of initiation of cigar products among youth, which has never before been reported. We conducted two separate analyses on never cigar product users: one that includes participants who had never heard of cigar products and another that includes only participants who had ever heard of cigar products. The analyses of youth who had never heard of cigar products at their first wave of PATH participation reinforced that their naiveté was a protective factor. We also used an interaction approach to assess the potential antagonistic association of perceptions of harmfulness and addictiveness on the age of initiation among youth who had ever heard of cigar products. Our study is unique in these ways.

We found that among youth who had ever heard of cigar products, youth who perceive cigar products as having low–medium harmfulness and low–medium addictiveness were at a higher risk of an earlier age of initiation of ever use and past 30–day use of cigar products. As cigar product use increases in popularity among youth, our findings support the need to focus and expand prevention and awareness campaigns around harmfulness and addictiveness of cigar products to curb cigar product initiation and prevent the risk of long–term health effects and lifelong nicotine addiction among US youth.

## Data availability statement

The datasets analyzed for this study (waves 1–4) are available from the Population Assessment of Tobacco and Health (PATH) Study [United States] Restricted–Use Files. Inter–university Consortium for Political and Social Research [distributor], 2020–06–24. https://doi.org/10.3886/ICPSR36231.v27. Researchers can apply for access to the restricted–use datasets from the Inter–university Consortium for Political and Social Research (ICPSR) at the University of Michigan. To access data in the Virtual Data Enclave (VDE), a Restricted Data Use Agreement (RDUA) must be established between the University of Michigan and the researcher's institution. Data are provided via ICPSR's VDE. For further information, please reference the VDE Guide to learn about the application process, about using the VDE, and how to request disclosure review of VDE output located here: https://www.icpsr.umich.edu/web/pages/NAHDAP/vde/index.html Obtaining results using the restricted–use datasets requires a disclosure process with protocols set by ICPSR. When a researcher logs on to the VDE, a virtual machine is launched on the researcher's own desktop but operates from a server at ICPSR. The virtual machine is isolated from the researcher's physical desktop computer – users cannot download or upload files or parts of files from or to the VDE; print VDE contents to a printer; or email, copy, or otherwise move files in or out of the VDE computing environment, either accidentally or intentionally. Results are only disclosed by ICPSR after programs have been checked for accuracy and results have been replicated. Requests to access these datasets should be directed to: https://www.icpsr.umich.edu/web/pages/NAHDAP/vde/index.html.

## Ethics statement

The studies involving human participants were reviewed and approved by Committee for the Protection of Human Subjects at the University of Texas Health Science Center at Houston with number HSC–SPH−17–0368. Written informed consent to participate in this study was provided by the participants' legal guardian/next of kin.

## Author contributions

BC contributed with formal analysis, data curation, investigation, software, writing, editing and review of manuscript. CS contributed with drafting, editing and reviewing the manuscript, help with the investigation and visualization of tables. MB contributed with conceptualization, methodology, resources, investigation, funding acquisition and writing and reviewing manuscript. AK contributed with drafting, editing, reviewing and interpretation of results. MH contributed with editing and reviewing the manuscript as well as interpretation of results. AP contributed with the conceptualization, methodology, formal analysis, resources, investigation, data curation, review and editing, supervision, project administration, visualization and funding acquisition. All authors contributed to the article and approved the submitted version.

## Funding

Research reported in this paper was supported by a grant awarded to AP [R01 CA234205] from the National Cancer Institute and the Food and Drug Administration (FDA) Center for Tobacco Products (CTP).

## Conflict of interest

Author MH is a consultant in litigation involving the vaping industry. The remaining authors declare that the research was conducted in the absence of any commercial or financial relationships that could be construed as a potential conflict of interest.

## Publisher's note

All claims expressed in this article are solely those of the authors and do not necessarily represent those of their affiliated organizations, or those of the publisher, the editors and the reviewers. Any product that may be evaluated in this article, or claim that may be made by its manufacturer, is not guaranteed or endorsed by the publisher.

## Author disclaimer

The content is solely the responsibility of the authors and does not necessarily represent the official views of the NIH or the FDA.
