# Supplementary material for: The association of perceptions of harmfulness and addictiveness on the age of initiation of cigar product use among youth: Findings from the Population Assessment of Tobacco and Health (PATH) study, 2013–2017
Source: Front Public Health. 2022 Oct 5;10:882434. doi: 10.3389/fpubh.2022.882434 (PMC9580780; doi:10.3389/fpubh.2022.882434)

**The association of perceptions of harmfulness and addictiveness on the age of initiation of cigar product use among youth: Findings from the Population Assessment of Tobacco and Health (PATH) study, 2013-2017.**

Baojiang Chen, Charles E. Spells, Meagan A. Bluestein, Arnold E. Kuk, Melissa B. Harrell, and Adriana Pérez

**Supplementary Tables and Figures**

**Supplementary Table 1. Hazard ratio (and 95% confidence intervals) of perceptions of harmfulness and addictiveness for each cigar outcome*.**

|  | **Ever Use** | **Past 30-Day Use** | **Fairly Regular Use** |
| --- | --- | --- | --- |
| **Univariate Analysis** | | | |
| **Perception of harmfulness** | | | |
| High | 1.00 | 1.00 | 1.00 |
| Low | **2.11 (1.78-2.49)** | **2.10 (1.67-2.64)** | **2.39 (1.16-4.94)** |
| Medium | **1.39 (1.24-1.56)** | **1.21 (1.04-1.42)** | 1.46 (0.86-2.47) |
| Don’t know | 1.58 (0.93-2.69) | 1.43 (0.61-3.37) | 1.84 (0.28-12.26) |
| Never heard | **0.66 (0.59-0.75)** | **0.67 (0.57-0.79)** | 0.53 (0.26-1.07) |
| **Perception of addictiveness** | | | |
| High | 1.00 | 1.00 | 1.00 |
| Low | **1.67 (1.41-1.99)** | **1.75 (1.39-2.20)** | 1.25 (0.56-2.80) |
| Medium | **1.41 (1.19-1.67)** | **1.34 (1.07-1.69)** | 0.61 (0.27-1.37) |
| Don’t know | 1.18 (0.88-1.59) | 1.28 (0.85-1.93) | 0.37 (0.03-5.10) |
| Never heard | **0.60 (0.54-0.68)** | **0.64 (0.54-0.76)** | **0.40 (0.21-0.74)** |
| **Sex** | | | |
| Female | 1.00 | 1.00 | 1.00 |
| Male | **1.73 (1.54-1.95)** | **1.97 (1.72-2.26)** | **1.63 (1.05-2.54)** |
| **Race** | | | |
| Non-Hispanic White | 1.00 | 1.00 | 1.00 |
| Non-Hispanic Black | 1.00 (0.82-1.22) | **1.26 (1.01-1.59)** | 1.30 (0.75-2.26) |
| Non-Hispanic Other^Ϯ^ | **0.63 (0.50-0.80)** | **0.64 (0.48-0.85)** | 0.78 (0.37-1.65) |
| Hispanic | **0.68 (0.59-0.78)** | **0.72 (0.61-0.85)** | 0.57 (0.31-1.06) |
| **Number of other tobacco products ever used** | | | |
| 0 | 1.00 | 1.00 | 1.00 |
| 1 | **2.28 (1.98-2.63)** | **2.18 (1.84-2.58)** | **2.09 (1.29-3.40)** |
| 2+ | **2.97 (2.54-3.48)** | **3.16 (2.60-3.83)** | **2.38 (1.34-4.22)** |
| **Multivariable analysis for perceptions of harmfulness** | | | |
| **Perception of harmfulness** | | | |
| High | 1.00 | 1.00 | 1.00 |
| Low | **1.87 (1.56-2.25)** | **1.83 (1.46-2.29)** | **2.08 (1.01-4.30)** |
| Medium | **1.26 (1.11-1.43)** | 1.09 (0.92-1.29) | 1.35 (0.79-2.31) |
| Don’t know | 1.51 (0.92-2.47) | 1.37 (0.60-3.12) | 1.66 (0.25-11.19) |
| Never heard | **0.71 (0.63-0.80)** | **0.72 (0.61-0.85)** | 0.56 (0.28-1.13) |
| **Sex** | | | |
| Female | 1.00 | 1.00 | 1.00 |
| Male | **1.79 (1.58-2.01)** | **2.01 (1.75-2.30)** | **1.63 (1.03-2.56)** |
| **Race** | | | |
| Non-Hispanic White | 1.00 | 1.00 | 1.00 |
| Non-Hispanic Black | 1.01 (0.83-1.23) | **1.32 (1.05-1.66)** | 1.31 (0.75-2.28) |
| Non-Hispanic Other^Ϯ^ | **0.76 (0.60-0.97)** | **0.78 (0.58-1.04)** | 0.94 (0.44-1.99) |
| Hispanic | **0.70 (0.61-0.79)** | **0.74 (0.62-0.87)** | 0.62 (0.33-1.16) |
| **Number of other tobacco products ever used** | | | |
| 0 | 1.00 | 1.00 | 1.00 |
| 1 | **2.22 (1.92-2.55)** | **2.14 (1.79-2.57)** | **1.95 (1.19-3.20)** |
| 2+ | **2.78 (2.36-3.27)** | **3.11 (2.55-3.78)** | **2.20 (1.24-3.90)** |
| **Multivariable analysis for perceptions of addictiveness** | | | |
| **Perception of addictiveness** | | | |
| High | 1.00 | 1.00 | 1.00 |
| Low | **1.34 (1.11-1.61)** | **1.35 (1.05-1.72)** | 1.00 (0.43-2.33) |
| Medium | **1.22 (1.02-1.46)** | 1.13 (0.89-1.43) | 0.53 (0.23-1.21) |
| Don’t know | 1.14 (0.85-1.54) | 1.26 (0.84-1.91) | 0.35 (0.02-4.87) |
| Never heard | **0.65 (0.57-0.73)** | **0.69 (0.58-0.82)** | **0.43 (0.23-0.79)** |
| **Sex** | | | |
| Female | 1.00 | 1.00 | 1.00 |
| Male | **1.77 (1.57-2.00)** | **1.99 (1.74-2.29)** | **1.67 (1.05-2.65)** |
| **Race** | | | |
| Non-Hispanic White | 1.00 | 1.00 | 1.00 |
| Non-Hispanic Black | 1.01 (0.82-1.23) | **1.31 (1.04-1.66)** | 1.37 (0.78-2.41) |
| Non-Hispanic Other^Ϯ^ | **0.74 (0.58-0.95)** | 0.77 (0.57-1.03) | 0.97 (0.46-2.05) |
| Hispanic | **0.68 (0.60-0.78)** | **0.73 (0.61-0.86)** | 0.61 (0.33-1.15) |
| **Number of other tobacco products ever used** | | | |
| 0 | 1.00 | 1.00 | 1.00 |
| 1 | **2.21 (1.92-2.54)** | **2.14 (1.78-2.56)** | **2.06 (1.24-3.42)** |
| 2+ | **2.78 (2.36-3.28)** | **3.08 (2.51-3.78)** | **2.37 (1.32-4.27)** |

*PATH Restricted file received disclosure to publish: April 22, 2021. United States Department of Health and Human Services. National Institutes of Health. National Institute on Drug Abuse, and United States Department of Health and Human Services. Food and Drug Administration. Center for Tobacco Products. Population Assessment of Tobacco and Health (PATH) Study [United States] Restricted-Use Files. ICPSR 36231-v13.AnnArbor, MI: Inter-university Consortium for Political and Social Research [distributor], November 5, 2019. <Https://doi.org/10.3886/ICPSR36231.v23>; ^Ϯ^Non-Hispanic Others include Asian, multi-race, etc.

**Supplementary Table 2. Hazard ratio (and 95% confidence intervals) of the interaction between perceptions of harmfulness and addictiveness *.**

|  | **Ever Use** | **Past 30-Day Use** | **Fairly Regular Use** |
| --- | --- | --- | --- |
|  |  |  |  |
| **Perception of harmfulness and addictiveness** | | | |
| High harm and high addictiveness | 1.00 | 1.00 | 1.00 |
| High harm and low-medium addictiveness | 1.16 (0.91-1.46) | 1.07 (0.81-1.41) | **0.24 (0.07-0.83)** |
| Low-medium harm and high addictiveness | **1.33 (1.15-1.53)** | 1.15 (0.95-1.40) | 1.36 (0.78-2.38) |
| Low-medium harm and low-medium addictiveness | **1.60 (1.36-1.89)** | **1.46 (1.14-1.86)** | 1.31 (0.66-2.61) |
| **Sex** | | | |
| Female | 1.00 | 1.00 | 1.00 |
| Male | **1.76 (1.56-2.00)** | **1.87 (1.62-2.16)** | 1.48 (0.90-2.43) |
| **Race** | | | |
| Non-Hispanic White | 1.00 | 1.00 | 1.00 |
| Non-Hispanic Black | 1.00 (0.81-1.25) | **1.34 (1.03-1.73)** | 1.48 (0.81-2.68) |
| Non-Hispanic Other^Ϯ^ | 0.91 (0.70-1.19) | 0.92 (0.64-1.31) | 0.84 (0.33-2.12) |
| Hispanic | 0.72 (0.62-0.82) | **0.77 (0.63-0.95)** | 0.72 (0.37-1.41) |
| **Number of other tobacco products ever used** | | | |
| 0 | 1.00 | 1.00 | 1.00 |
| 1 | **1.95 (1.68-2.26)** | **1.85 (1.52-2.27)** | **1.78 (1.06-3.00)** |
| 2+ | **2.53 (2.10-3.05)** | **2.73 (2.17-3.43)** | **2.19 (1.26-3.82)** |

*PATH Restricted file received disclosure to publish: April 22, 2021. United States Department of Health and Human Services. National Institutes of Health. National Institute on Drug Abuse, and United States Department of Health and Human Services. Food and Drug Administration. Center for Tobacco Products. Population Assessment of Tobacco and Health (PATH) Study [United States] Restricted-Use Files. ICPSR 36231-v13.AnnArbor, MI: Inter-university Consortium for Political and Social Research [distributor], November 5, 2019. <Https://doi.org/10.3886/ICPSR36231.v23>

^Ϯ^Non-Hispanic Others include Asian, multi-race, etc.

Figure 1. Estimated hazard function for age of initiation of ever cigar use, stratified by perceptions of harmfulness and addictiveness


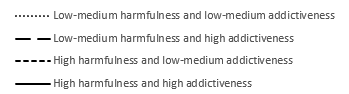


Figure 2. Estimated hazard function for age of initiation of past 30-day cigar use, stratified by perceptions of harmfulness and addictiveness


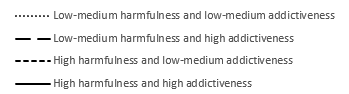


Figure 3. Estimated hazard function for age of initiation of fairly regular cigar use, stratified by perceptions of harmfulness and addictiveness


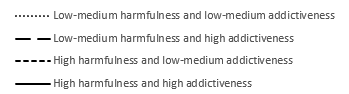

Supplement: Supplementary file 1 [file Data_Sheet_1.docx]
